# Sequence Analysis and Comparison of TCTP Proteins from Human Protozoan Parasites

**DOI:** 10.1007/s11686-022-00521-9

**Published:** 2022-02-09

**Authors:** Francisco Alejandro Lagunas-Rangel

**Affiliations:** Department of Surgical Sciences, Functional Pharmacology and Neuroscience, Uppsala University, Husargatan 3, BMC Box 593, 75124 Uppsala, Sweden

**Keywords:** Histamine releasing factor, Phylogenetic analysis, Flexible loop, Dominant negative mutant

## Abstract

**Purpose:**

Translational controlled tumor protein (TCTP) is a functionally important protein in most eukaryotes because it participates in a wide variety of processes, the most representative being proliferation, differentiation, histamine release, cell death, protein synthesis and response to stress conditions. In the present work, we analyze the sequence, structure and phylogeny of TCTP orthologs in a group of human parasitic protozoan species.

**Methods:**

The complete sequences of TCTP orthologs in protozoan parasites were identified with the NCBI BLAST tool in the database of the EuPathDB Bioinformatics Resource Center. The sequences were aligned and important regions of the protein were identified, and later phylogenetic trees and 3D models were built with different bioinformatic tools.

**Results:**

Our results show evolutionarily and structurally conserved sites that could be exploited to create new therapeutic strategies given the increase in the number of strains resistant to current drugs.

**Conclusion:**

TCTP orthologs in protozoan parasites have been little studied but have been shown to be important in parasite growth, proliferation, reproduction, and response to changes in the environment. For all this, TCTP can be considered as a possible therapeutic target.

**Supplementary Information:**

The online version contains supplementary material available at 10.1007/s11686-022-00521-9.

## Introduction

Translational controlled tumor protein (TCTP), also called fortilin, P23 or histamine releasing factor (HRF), is a small multifunctional protein that is ubiquitous in eukaryotes [1]. This protein participates in a wide variety of key regulatory processes, such as control of proliferation and growth, pluripotency and differentiation, regulation of cell death, protein synthesis and degradation, the response to stress conditions and autophagy, microtubule stabilization, and histamine release, among others [2].

Particularly in human parasites, little is currently known about the functions and processes that involve TCTP orthologs, but some studies have shown that they collaborate in parasite growth, proliferation, reproduction and response to stress conditions, which is why this protein has been suggested as a possible therapeutic target [3–5]. *Toxoplasma gondii* has been reported to require its TCTP ortholog for rapid growth and to maintain its intracellular development [3]. Moreover, TCTP has been shown to act as an antioxidant and heat shock protein with chaperone activity in some parasites [6–8] and can also interact and sequester antiparasitic drugs, thus contributing to the development of resistant strains [9–11]. TCTP has been reported to be present in the plasma of *Plasmodium falciparum*-infected patients at concentrations between 2.4 and 6.9 µg/mL, indicating that these parasites secrete this protein [12]. In the same way, it has also been shown that other parasites also release this protein into the environment and it has been suggested that the TCTP protein secreted by the parasites could block the normal immune response by acting as a dominant negative mutant of human TCTP [4, 5, 13–15]. Notably, immunization of BALB/c mice with the purified recombinant TCTP protein from *Plasmodium falciparum* or *Plasmodium yoelii* reduced the parasitaemia generated during the early stages of *Plasmodium yoelii* or *Plasmodium chabaudi* infection and increased survival [16]. Likewise, a transient reduction in parasitemia was observed in OF1 mice infected with *Trypanosoma brucei gambiense* that were previously immunized with the parasite’s TCTP protein [13]. The production of anti-TCTP antibodies in the immunized mice in both assays indicates that the protein is capable of inducing an adaptive immune response [13, 16]. Furthermore, TCTP of some parasites has been shown to play a role in the development of allergic inflammatory responses in infected patients [14, 17], stimulating the release of histamine from basophils and IL-8 from eosinophils [12]. For all this, it is thought that TCTP orthologs in parasites could act in two different areas, one involved in the regulation of host cytokine signaling and the other in the maintenance of parasite homeostasis and its response to conditions of stress [15].

The proposal of the present work was to analyze, from different human parasitic protozoan species, the sequence, structure and phylogeny of TCTP orthologs to identify notable characteristics that could be exploited in the development of new therapeutic strategies.

## Materials and Methods

### Database Screening

The orthologues of the human TCTP protein in protozoan parasites were found using the NCBI BLAST tool (with predetermined search parameters) in the database of the EuPathDB Bioinformatics Resource Center [18]. The complete sequences of the TCTP orthologs were compiled and included the following species: *Homo sapiens* (P13693), *Cryptosporidium parvum* (CPATCC_001190), *Cryptosporidium hominis* (Chro.30228), *Toxoplasma gondii* (TGME49_251680), *Sarcocystis neurona* (SN3_51680), *Cyclospora cayetanensis* (cyc_00965), *Acanthamoeba castellanii* (ACA1_069360 and ACA1_030760), *Entamoeba histolytica* (EHI_021360), *Naegleria fowleri* (FDP41_001128, NF0012290, FDP41_001927 and NF0009160), *Giardia duodenalis* (GL50803_6242 and DHA2_6242), *Plasmodium falciparum* (PF3D7_0511000), *Trypanosoma brucei* (Tb927.8.6750 and Tb927.8.6760), *Trypanosoma cruzi* (TcCLB.506207.50), *Leishmania major* (LmjF.24.1510 and LmjF.24.1500), *Leishmania infantum* (LINF_240020500 and LINF_240020600), *Leishmania donovani* (LdBPK_241560.1), *Leishmania braziliensis* (LbrM.24.1350), *Leishmania mexicana* (LmxM.24.1500a and LmxM.24.1500), and *Trichomonas vaginalis* (TVAG_180840).

### Sequence Analysis, Alignments, Phylogenetic Analysis, and Protein Modeling

The sequences of the DDP4 proteins from all the organisms studied were analyzed using InterPro [19]. All the sequences obtained were aligned with the MEGA11 software [20] using the MUSCLE algorithm and then manually edited after visual inspection. Conservation calculations were performed with CLC Genomics Workbench 21 (QIAGEN Digital Insights). The aligned sequences were used to construct the phylogenetic trees using maximum likelihood and minimum evolution methods. The 3D models of the TCTP proteins were predicted with SWISS-MODEL [21], and illustrations were made using UCSF Chimera software [22].

## Results

### Sequence Analysis

**Fig. 1:**
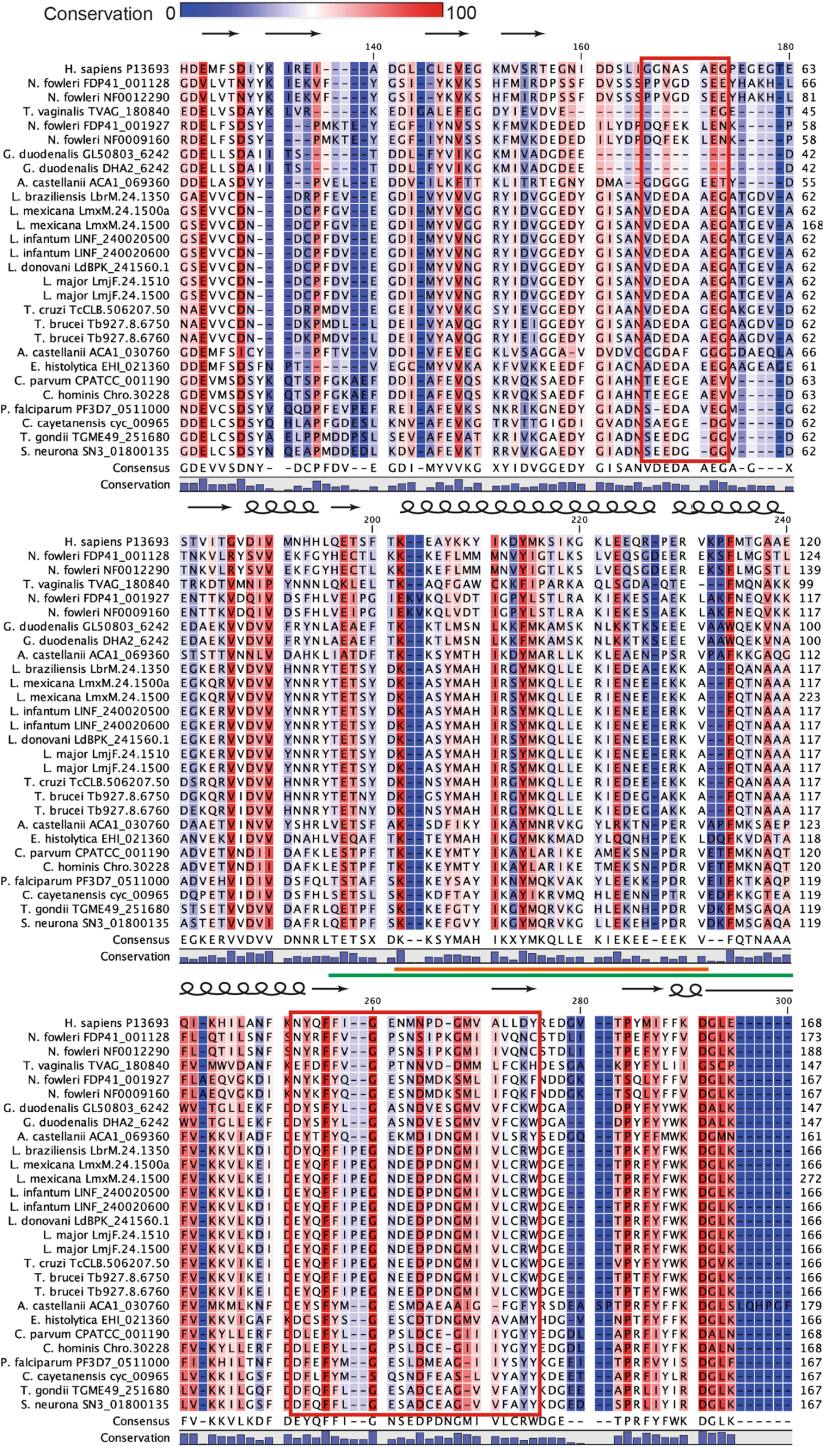
Partial alignment of the sequences of TCTP orthologs identified in protozoan parasites. Consensus was obtained on all available sequences. TCTP1 and TCTP2 fingerprints are identified by red boxes. The calcium (orange) and microtubule (green) binding regions are underlined. Letters with a red background indicate conserved residues. The predicted secondary structure corresponds to human TCTP. β-sheets are represented as arrows, while α-helices are represented as a spiral line

After obtaining the complete sequences of the TCTP orthologs identified in the protozoan parasites, we performed an alignment (Supplementary Fig. 1) that showed an average identity of 30.9% and an overall similarity of 49.08% with respect to the human protein (Table 1). There was an overall identity of 38.19% among all proteins. The orthologist ACA1_069360 from *A. castellanii* was the one that had the greatest identity and similarity with the human protein. Protein sizes were around 151 and 229 amino acids (*T. vaginalis* and *N. fowleri* proteins, respectively) and the two protein fingerprints (TCTP1 and TCTP2) characteristic of this protein in all species were identified (Fig. 1). The TCPT1 fingerprint had 55.62% identity, while the TCTP2 fingerprint had 64.95%. Considering the positions of our alignment, the most conserved residues were M107 (96%), E123 (93%), D127 (89%), V186 (89%), K202 (93%), Y214 (89%), K234 (93%), F255 (100%), G260 (96%), P284 (93%) and D291 (96%), and L293 (89%).

**Table 1:** Identity and similarity of TCTP orthologues in protozoan parasites compared to human protein

Species	UniProtKB^a^	Size^b^	I^c^	S^d^
*Cryptosporidium parvum*	CPATCC_001190	172	30.51	50.85
*Cryptosporidium hominis*	Chro.30228	172	30.51	50.85
*Toxoplasma gondii*	TGME49_251680	171	35.03	56.50
*Sarcocystis neurona*	SN3_51680	171	32.77	54.80
*Cyclospora cayetanensis*	cyc_00965	171	31.07	50.85
*Acanthamoeba castellanii*	ACA1_069360	165	39.88	57.80
	ACA1_030760	191	35.75	52.85
*Entamoeba histolytica*	EHI_021360	170	36.42	57.23
*Naegleria fowleri*	FDP41_001128	229	21.19	33.05
	NF0012290	244	19.92	31.08
	FDP41_001927	171	27.78	46.67
	NF0009160	171	27.78	46.67
*Giardia duodenalis*	GL50803_6242	151	28.74	43.68
	DHA2_6242	151	28.74	43.68
*Trichomonas vaginalis*	TVAG_180840	151	26.16	39.53
*Plasmodium falciparum*	PF3D7_0511000	171	33.14	53.71
*Trypanosoma brucei*	Tb927.8.6750	170	32.96	53.63
	Tb927.8.6760	170	31.43	55.43
*Trypanosoma cruzi*	TcCLB.506207.50	170	30.43	51.09
*Leishmania major*	LmjF.24.1510	170	33.70	51.63
	LmjF.24.1500	170	33.70	51.63
*Leishmania infantum*	LINF_240020500	170	33.15	51.63
	LINF_240020600	170	33.15	51.63
*Leishmania donovani*	LdBPK_241560.1	170	33.15	51.63
*Leishmania braziliensis*	LbrM.24.1350	170	31.84	52.51
*Leishmania mexicana*	LmxM.24.1500a	170	33.15	50.54
	LmxM.24.1500	276	22.34	34.04
Average		177.70	30.90	49.08

^a^Access number in the UniProt Knowledgebase

^b^Number of amino acids

^c^I (Identity) values expressed in percentage (%)

^d^S (Similarity) values expressed in percentage (%)

### Phylogenetic Analysis

**Fig. 2:**
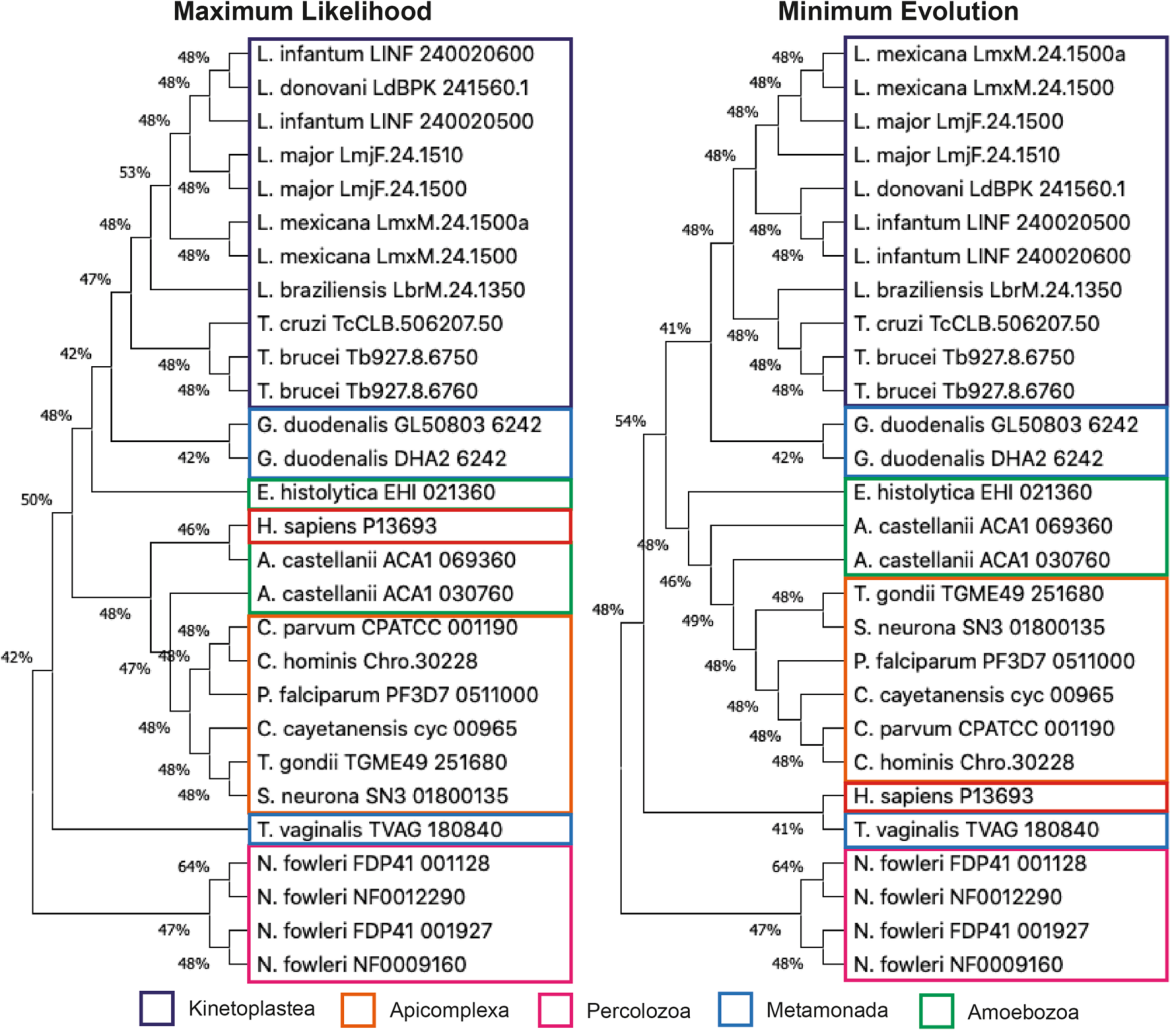
Phylogenetic trees of TCTP orthologs in protozoan parasites. The evolutionary distances between the species were calculated with the algorithms of maximum likelihood and minimum evolution. The percentage of replicate trees in which the associated taxa clustered together in the bootstrap test are shown next to the branches. The sequence of human TCPT was added for comparative purposes

The sequence alignment of TCTP orthologs was used to build phylogenetic trees using two methods to have different perspectives, a character-based method such as maximum likelihood and a distance-based method such as minimum evolution (Fig. 2). Both methods constructed similar phylogenetic trees, placing the *N. fowleri* orthologs as the most ancestral proteins, while the orthologues of organisms in the *Kinetoplastea* class were the most evolutionarily advanced. Both trees separated TCTP orthologs from *G. duodenalis* and *T. vaginalis*, although both species belong to the *Metamonada* phylum. The *T. vaginalis* ortholog was closer to those of *N. fowleri*, while the *G. duodenalis* orthologs to those of the *Kinetoplastea* class. The orthologs of the species of the subphylum *Apicomplexa* and of the phylum *Amoebozoa* were placed in the middle of the proteins of *G. duodenalis* and *T. vaginalis*. The trees had differences when placing the human TCTP protein, the algorithm of maximum likelihood placed it evolutionarily close to the phylum *Amoebozoa*, particularly of *A. castellanii* orthologue ACA1_069360, while the algorithm of minimum evolution placed it close to *T. vaginalis* orthologue.

### Structural Analysis

**Fig. 3:**
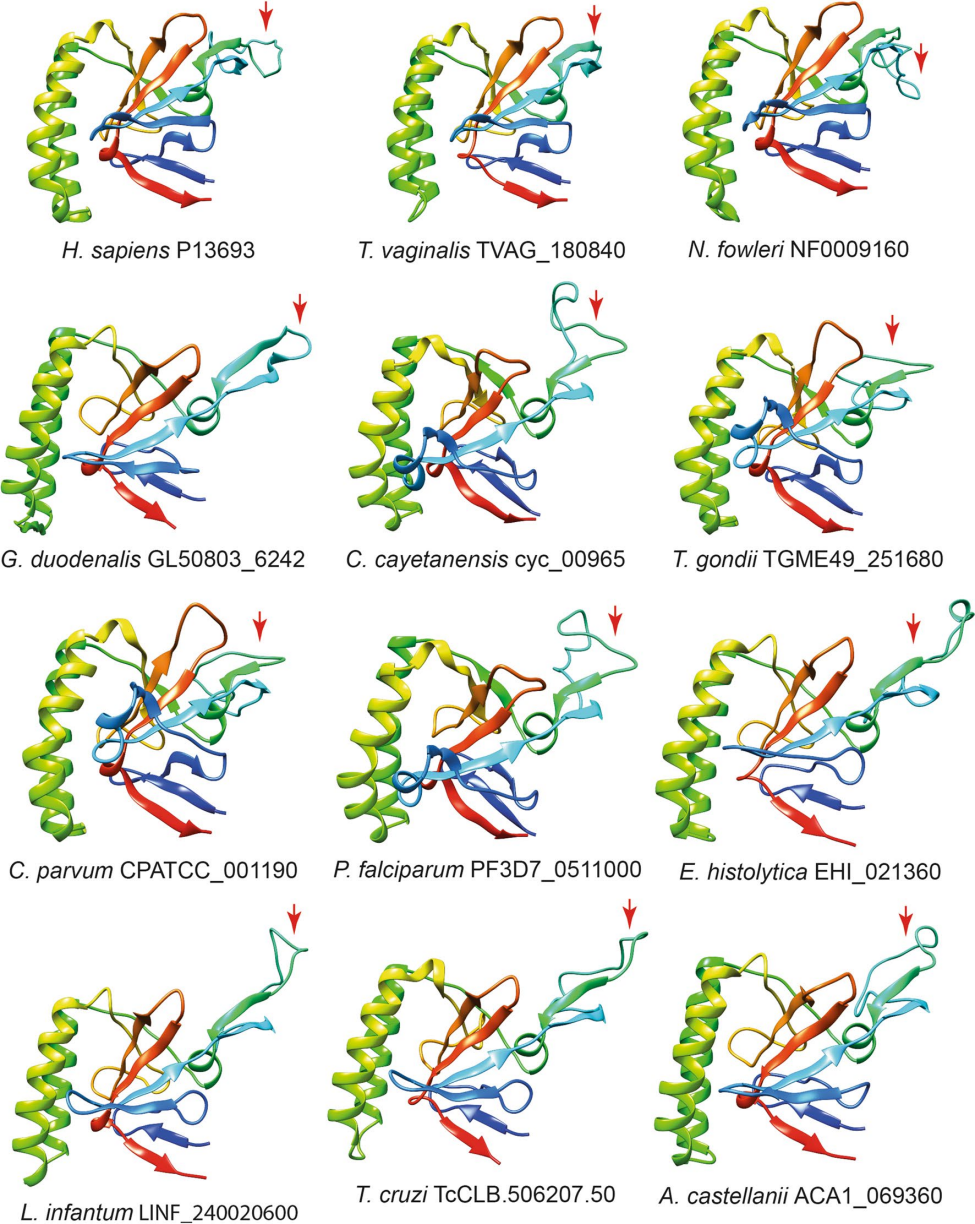
Comparison of predicted three-dimensional structures of TCTP orthologs in protozoan parasites. Structures are represented as ribbons and chains. Note the high level of structural similarity exhibited between these representative TCTPs. The flexible loop, which is the region with the most notable differences between TCTP orthologs studied, is indicated by a red arrow

We constructed 3D models of one of the TCTP orthologs in each organism using the human protein as a point of comparison (Fig. 3). First, we realized that the number and arrangement of β-sheets and α-helices is maintained in all species, only with variations in the size of these secondary structures. The tertiary structure was also similar in all the predicted proteins, the main difference being the size of the bridge between the fifth and sixth β sheet (flexible loop/TCTP1), which tends to be larger in the parasites studied compared to human protein.

## Discussion

Given the functional relevance of TCTP in eukaryotes and its possible use as a therapeutic target against protozoan parasites, we carried out an analysis of this protein from several aspects. A moderate identity (38.19%) was found when aligning the sequences of all the orthologues studied and the TCTP1 and TCPT2 fingerprints were identified in all of them. Among the most conserved residues that we found in the protozoan parasites studied was E123 (corresponding to residue 12 of human TCTP), which previous reports showed that it is an important residue for the binding of TCTP to RHEB (RAS homologue enrichment in brain) GTPase to activate TOR (Target of rapamycin) [23]. In contrast, residues S46 and S64 of human TCTP (residues 163 and 181 of our alignment), which are subject to phosphorylation by PLK, are not conserved in parasites and are only found in mammalian TCTPs [24, 25]. Interestingly, the V186 residue of our alignment that was highly conserved in parasites, in humans (residue 69) is changed by a glycine that participates in the binding of TCTP with the PIM-3 (Provirus integrating site Moloney murine leukemia virus 3) kinase [26]. The conservation of K202 (residue 85 of human TCTP), F255 (residue 114 of human TCTP), G260 (residue 137 of human TCTP) and P284 (residue 158 of human TCTP) may be due to their participation in the homodimerization of the protein [27]. For residue D291 (residue 165 of human TCTP), its participation in the binding of TCTP to G proteins has been suggested [15]. In contrast, the residues corresponding to L78 (residue 195 in our alignment) and Q134 (residue 254 in our alignment) of human TCTP, which are also important for the binding of this protein to G proteins [28], were poorly preserved in parasites. L78 was only present in 52% of the protozoan parasites, particularly in parasites of the *Kinetoplastea* class, being mainly changed by a tyrosine, whereas Q134 was only present in 48% and was exchanged for different amino acids. The latter reinforces the theory that parasite TCTPs can act as dominant negative mutants. It has been mentioned previously that *Plasmodium* TCTP, which is more efficiently incorporated into B cells than its human counterpart [29], induces the release of histamine, but could block the activation of B cells by not being able to interact with G proteins and, therefore, the parasite protects itself, to some extent, from the host’s immune system [15].

Regarding our phylogenetic analysis, the clusters generated are largely in agreement with the proposed phylogeny of eukaryotes (Fig. 2) [30, 31]. The phylogenetic trees identified the TCTP orthologs in the amoebo-flagellate *N. fowleri* as the most ancestral proteins. *N. fowleri* belongs to the *Heterolobosea* clade that diverged from other eukaryotic lineages over a billion years ago [32]. Meanwhile, the phylum *Metamonada* has also been identified as one of the oldest, and particularly the flagellated protozoan *G. duodenalis* has been recognized as one of the closest to ancestral eukaryotes, although there is still much debate as to the evidence supporting this claim [33–36]. *N. fowleri, T. vaginalis* and *G. duodenalis* belong to the *Excavata* sub-kingdom. The members of the *Apicomplexa* subphylum belonging to the SAR supergroup (which includes *Stramenopiles*, *Alveolata* and *Rhizaria*) and also the members of the *Amoebozoa* phylum are considered evolutionarily more advanced that the members of the *Excavata* sub-kingdom [30, 31]. The evolutionary position of the *Kinetoplastea* class is not yet well established, but since parasitic kinetoplastids have arisen from non-pathogenic free-living ancestors, multiple processes must have occurred before this step could be taken [37].

Structural analysis of TCTP orthologs predicted a highly conserved secondary and tertiary structure (Fig. 3). Furthermore, an increase in flexible loop size was observed in most TCTP orthologs compared to human protein. Although we do not know the functional reason for this change, it is known that the flexible loop is critical to promote cytokine release and the limitation of the flexibility of this region by disulfide bonds is required [38]. Notably, this area has also served as a binding site for potential TCTP inhibitors (Brompheniramine, Sertraline, ZINC19364226, ZINC19361042, ZINC03831041, ZINC12657067, ZINC12657071, ZINC12863423, ZINC08918508) [39]. This opens up the option of creating new therapeutic strategies against protozoan parasites taking TCTP as a target, which would also take advantage of the differences in this region compared to human protein. *P. falciparum* TCTP has been suggested to be a potential target for artemisinin, one of the most effective antimalarial drugs, and some residues involved in binding have been described. The C14 residue (position 115 of our alignment) of *P. falciparum* TCTP and other species of the subphylum *Apicomplexa* has been proposed as one of the possible artemisinin alkylation sites and could serve as an electron donor during endoperoxide activation [40, 41]. However, in species of the *Kinetoplastea* class, this cysteine is found in position 15 (116 of our alignment) and in the other species of protozoan parasites, this residue is replaced by hydrophobic amino acids (phenylalanine, valine, alanine or leucine) (Fig. 1) that have been shown to completely inhibit binding to artemisinin [40]. The F8A mutation (position 119 of our alignment) also significantly decreased the binding of the *P. falciparum* TCTP protein to artemisinin [40], which is important since in human and most other protozoan parasites, it is replaced by a leucine or isoleucine (Supplementary Fig. 1). D11A or F18A mutations (positions 122 and 129 of our alignment) had only a small effect on artemisinin binding [40]. Furthermore, the regions corresponding to residues 19–46 (130–154 in our alignment), 108–134 (229–255 in our alignment) and 140–163 (265–290 in our alignment) have been reported to be covalently modified by artemisinin radicals [42]. Another thing that has to be considered when designing TCTP inhibitor drugs is that it is a dynamic molecule and in a study where a series of putative compounds inhibiting human TCTP were found [43], the amino acids most involved in the interaction with the chemicals were K34, M35, V36, G69, V70, D71, I72 and P158 (residues K151, M152, V153, G195, V196, D197, I198, P283 in our alignment).

In conclusion, we can say that TCTP orthologues have important functions in protozoan parasites, they maintain a moderate identity between them, but their structure is quite conserved, evolutionarily they follow the proposed phylogeny of eukaryotes and the changes in the flexible loop could be used in the creation of new antiparasitic drugs.

## Supplementary Information

Below is the link to the electronic supplementary material.

**Supplementary Figure 1. Complete alignment of the sequences of TCTP orthologs identified in protozoan parasites.** Consensus was obtained on all available sequences. TCTP1 and TCTP2 fingerprints are identified by red boxes. The calcium (orange) and microtubule (green) binding regions are underlined. Letters with a red background indicate conserved residues. The predicted secondary structure corresponds to human TCTP. β-sheets are represented as arrows, while α-helices are represented as a spiral line. (TIF 33835 KB)
