# Supplementary figures and images for: Sequence Analysis and Comparison of TCTP Proteins from Human Protozoan Parasites
Source: Acta Parasitol. 2022 Feb 9;67(2):1024–31. doi: 10.1007/s11686-022-00521-9 (PMC9165267; doi:10.1007/s11686-022-00521-9)

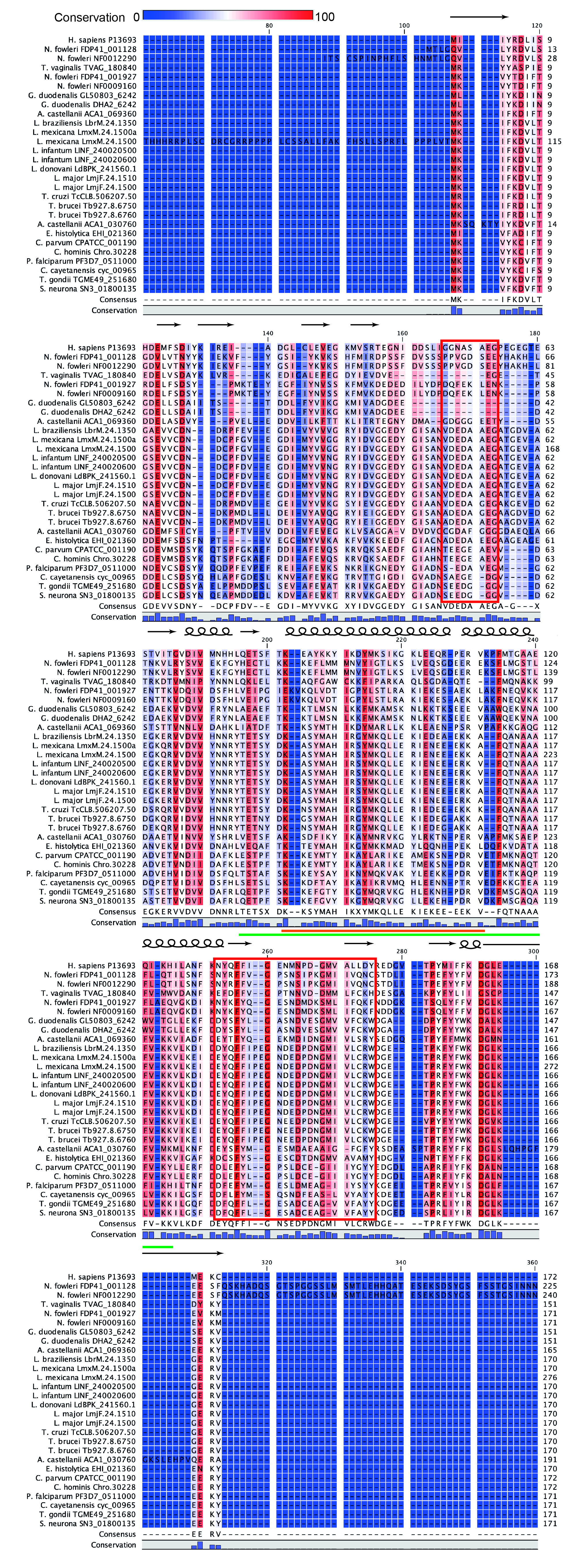

Supplement: Supplementary file 1 — Supplementary Figure 1. Complete alignment of the sequences of TCTP orthologs identified in protozoan parasites. Consensus was obtained on all available sequences. TCTP1 and TCTP2 fingerprints are identified by red boxes. The calcium (orange) and microtubule (green) binding regions are underlined. Letters with a red background indicate conserved residues. The predicted secondary structure corresponds to human TCTP. β-sheets are represented as arrows, while α-helices are represented as a spiral line. (TIF 33835 KB) [file 11686_2022_521_MOESM1_ESM.tif]
